# Autopsy findings in COVID-19-related deaths: a literature review

**DOI:** 10.1007/s12024-020-00310-8

**Published:** 2020-10-07

**Authors:** Aniello Maiese, Alice Chiara Manetti, Raffaele La Russa, Marco Di Paolo, Emanuela Turillazzi, Paola Frati, Vittorio Fineschi

**Affiliations:** 1 Department of Surgical Pathology, Medical, Molecular and Critical Area, Institute of Legal Medicine, University of Pisa, 56126 Pisa, PI Italy; 2 Department of Anatomical, Histological, Forensic and Orthopedic Sciences, Sapienza University of Rome, Viale Regina Elena 336, 00161 Rome, RM Italy

**Keywords:** COVID-19, Autopsy, Findings, Pathophysiology

## Abstract

Although many clinical reports have been published, little is known about the pathological post-mortem findings from people who have died of the novel coronavirus disease. The need for postmortem information is urgent to improve patient management of mild and severe illness, and treatment strategies. The present systematic review was carried out according to the Preferred Reporting Items for Systematic Review (PRISMA) standards. A systematic literature search and a critical review of the collected studies were conducted. An electronic search of PubMed, Science Direct Scopus, Google Scholar, and Excerpta Medica Database (EMBASE) from database inception to June 2020 was performed. We found 28 scientific papers; the total amount of cases is 341. The major histological feature in the lung is diffuse alveolar damage with hyaline membrane formation, alongside microthrombi in small pulmonary vessels. It appears that there is a high incidence of deep vein thrombosis and pulmonary embolism among COVID-19 decedents, suggesting endothelial involvement, but more studies are needed. A uniform COVID-19 post-mortem diagnostic protocol has not yet been developed. In a time in which international collaboration is essential, standardized diagnostic criteria are fundamental requirements.

## Introduction

The outbreak of the new SARS-CoV-2 (severe acute respiratory syndrome coronavirus 2) infection has spread all over the world [1], and on 11 March 2020, the World Health Organization declared it a pandemic [2]. COVID-19 (coronavirus disease 2019) has become a challenge for all health care authorities due to an increasing number of severely ill patients, which overloads intensive care units.

SARS-CoV-2 infection causes the release of a significant amount of pro-inflammatory cytokines that aggravate interstitial pneumonia and acute respiratory distress syndrome (ARDS) [3–6]. This clinical picture evolves into viral sepsis with prominent hypercoagulability and multiorgan dysfunction [7–11].

Autopsy findings are crucial to gaining a better understanding of how this infection affects the human body, similar to how these findings are important to understanding other infectious diseases [12–14]. Histopathological evidence of damage to the surface layers of airway epithelial cells and massive lung involvement with diffuse alveolar damage (DAD) and microvascular thrombi have been reported [15, 16].

The aim of this paper is to collect the currently available pathological data on COVID-19 and review the international literature on the postmortem findings of patients with COVID-19. A distinction between minimally invasive autopsy and complete autopsy is also made.

## Methods

The present systematic review was carried out according to the Preferred Reporting Items for Systematic Review (PRISMA) standards [17]. A systematic literature search and a critical review of the collected studies were conducted. An electronic search of PubMed, Science Direct Scopus, Google Scholar, and Excerpta Medica Database (EMBASE) from database inception to June 2020 was performed. The search terms were “COVID-19”, "SARS-CoV-2", "autopsy", “postmortem”, “biopsy”, and “histology” in the title, abstract, and keywords. The bibliographies of all located papers were examined and cross-referenced to further identify relevant literature. A methodological appraisal of each study was conducted according to the PRISMA standards, including an evaluation of bias. The data collection process included study selection and data extraction. Three researchers (RLR, PF, and MDP) independently examined the papers with titles or abstracts that appeared to be relevant and selected those that analyzed postmortem COVID-19 findings. Disagreements concerning eligibility among the researchers were resolved by consensus. Preprint articles were included. Data extraction was performed by two investigators (AM, ACM) and verified by other investigators (VF, ET). This study was exempt from institutional review board approval, as it did not involve human subjects. Only papers in English were included in the search, except for one study in which only the abstract was available in English.

## Results

**Fig. 1:**
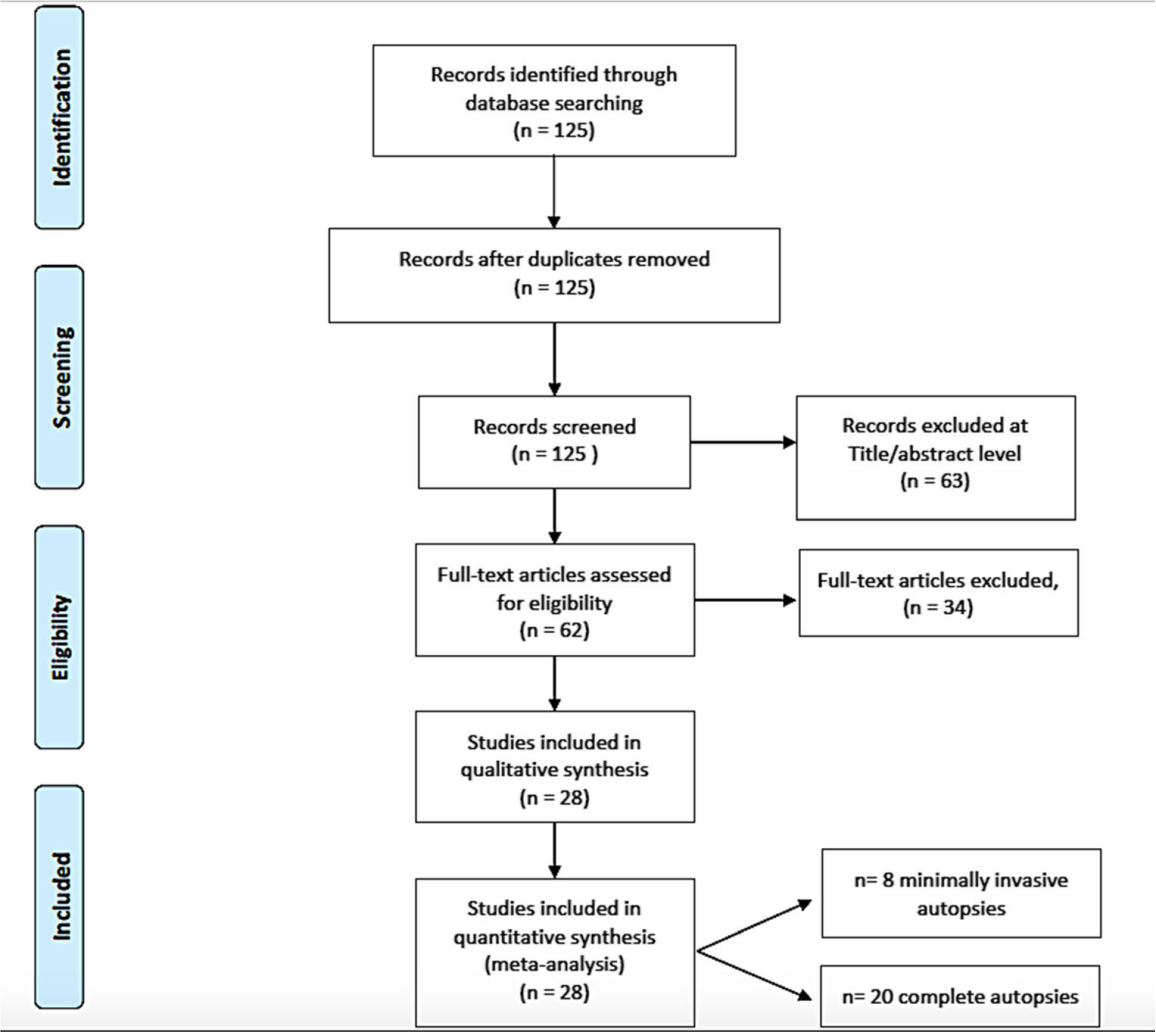
An appraisal based on titles and abstracts as well as a hand search of reference lists was carried out. The resulting 125 references were screened to exclude duplicates, which left 48 articles for further consideration. In addition, non-English papers were excluded and the following inclusion criteria were used: (1) original research articles, (2) reviews and mini-reviews, and (3) case report/series. These publications were carefully evaluated taking into account the main aims of the review. This evaluation left 28 scientific papers (8 concerning minimally invasive autopsies, 20 concerning complete autopsies), distributed as original research articles, case report/series, reviews, and mini-reviews

A review of the titles and abstracts as well as a manual search of the reference lists were carried out. The reference lists of all identified articles were reviewed to find missed literature. This search identified 125 articles, which were then screened based on their abstract. The resulting 125 reference lists were screened to exclude duplicates, which left 48 articles for further consideration. In addition, non-English papers were excluded, and the following inclusion criteria were used: (1) original research articles, (2) reviews and mini-reviews, and (3) case reports/series. These publications were carefully evaluated, taking into account the main aims of the review. This evaluation left 28 scientific papers (8 concerning minimally invasive autopsies, and 20 concerning complete autopsies) comprising original research articles, case reports/series, reviews, and mini-reviews. Figure 1 illustrates our search strategy. We divided the results into two groups: minimally invasive autopsies and complete autopsies (Table 1).

**Table 1:** Review of the literature on COVID-19 related-death autopsies, what kind of examination and analysis has been performed

	N° of Cases	Complete autopsy	Minimally invasive autopsy	Histology	Immunohistochemistry	Electron microscopy	Post-mortem imaging
Xu et al	1	-	1	1	Not reported	Not reported	Not reported
Zhang et al	1	-	1	1	1	Not reported	Not reported
Dolhnikoff et al	10	-	10	10	Not reported	Not reported	Not reported
Yao et al	3	-	3	3	3	3	Not reported
Tian et al	4	-	4	4	1 (in a case with CLL)	Not reported	Not reported
Duarte-Neto et al	10	-	10	10	10	Not reported	Not reported
Ramon y Cajal Hospital	1	-	1	1	1	Not reported	Not reported
Magro et al	2 (5 including in life biopsies)	-	2	2	2	Not reported	Not reported
Su et al	26	26	-	26	26	26	Not reported
Barton et al	2	2	-	2	2	Not reported	2 (full body a-p radiographs)
Grimes et al	2	2	-	2	Not reported	2	Not reported
Varga et al	2 (3 including 1 intestine resection)	2	-	3	Not reported	1	Not reported
Bradley et al	12	5	7	12	Not reported	12	Not reported
Paniz-Mondolfi et al	1	Not specified	Not specified	1	Not reported	1	Not reported
Lacy et al	1	1	-	1	Not reported	Not reported	Not reported
Konopka et al	1	1	-	1	Not reported	Not reported	Not reported
Prilutskiy et al	4	4	-	4	4	Not reported	Not reported
Yan et al	1	1	-	1	1	1	Not reported
Fitzek et al	1	1	-	1	Not reported	Not reported	1 (PMCT)
Edler et al	80	80	-	12	Not reported	Not reported	80 (PMCT)
Bryce et al	67	67	-	25	Not specified	Not specified	Not reported
Menter et al	21	17	4	21	11	2	Not reported
Remmelink et al	17	17	-	17	17	Not reported	Not reported
Wichmann et al	12	12	-	12	12	12 (only lung samples)	10 (PMCT)
Schaller et al	10	10	-	10	Not reported	Not reported	Not reported
Aguiar et al	1	1	-	1	1	Not reported	1 (PMCT)
Fox et al	10	10	-	10	10	10	Not reported
Carsana et al	38	38	-	38	On the most representative areas of randomly selected cases	10	Not reported
Total	341	297	44	232	101	80	94 (2 RX, 92 PMCT)

### Minimally invasive autopsies

As previously mentioned, the first work about the pathological findings of COVID-19 were based on biopsies and partial autopsies.

In February 2020, Xu et al. [15] described the case of a man who experienced 14 days of progressive respiratory symptoms and died due to sudden cardiac arrest during an episode of respiratory failure (oxygen saturation 60%). Core biopsies were conducted, and the lung, heart, and liver were sampled. The lungs showed early DAD with hyaline membranes and edema, the interstitium was infiltrated by lymphocytes, and pneumocytes manifested cytopathic changes (multinucleated syncytial cells), suggesting viral damage. No peculiar alterations of the myocardium were observed, except moderate interstitial mononuclear infiltrate. The liver tissue was characterized by microvesicular steatosis, a nonspecific feature that could not be directly correlated to the virus. The authors suggested that the overactivation of Tcells could be partially responsible for the immune system damage.

Zhang et al. [16] performed postmortem transthoracic needle biopsies of the lungs in a 72-year-old patient who died of respiratory insufficiency due to COVID-19. Lung tissue showed organizing DAD with fibrinous exudate into the alveoli and chronic inflammatory infiltrates and fibrosis of the interstitium. SARS-CoV-2-specific immunostaining revealed viral particles in the alveolar epithelium that were almost undetectable on the interstitium and vessel walls.

Dolhnikoff et al. [18] performed ultrasound-based minimally invasive autopsies of ten deceased patients with COVID-19 in São Paulo. The authors sampled the brain, lungs, heart, liver, spleen, kidneys, skeletal muscle, and skin. Histology revealed diffuse exudative and proliferative DAD alongside foci of alveolar hemorrhage. There was also little lymphocytic infiltration, and the epithelium of the alveoli and small airways showed viral cytopathic damage. Fibrin microthrombi were observed in small pulmonary arterioles of the lungs, glomeruli, and derma. In addition, there were many megakaryocytes within the pulmonary capillaries.

Li et al. [19] analyzed the three-dimensional histology reconstruction obtained from lung tissue samples of patients who died because of COVID-19. Their work emphasized the presence of megakaryocytes alongside fibrin aggregates in the pulmonary small vessels and viral cytopathic changes in pneumocytes.

In May 2020, Yao et al. [20] published a case series regarding minimally invasive autopsies of three COVID-19 decedents. Unfortunately, only the abstract of the paper is available in English. Nevertheless, it was included in our study as it could be useful for our purpose. The authors found hyaline membrane formation into the alveoli, with serous and fibrin exudate, and inflammatory infiltration was mainly represented by macrophages and lymphocytes (CD4-positive Tcells). Few multinucleated giant cells were also present. A peculiar finding was congestion and edema of the lung capillaries with modest infiltration of monocytes and lymphocytes and hyaline thrombi in the lumen. Focal hemorrhages were also observed. Immunohistochemistry and PCR (Polymerase Chain Reaction) analysis confirmed the presence of SARS-CoV-19 in the macrophages and alveolar epithelia.

The histopathological findings of lung, liver, and heart core biopsies conducted on four deceased COVID-19 patients were described by Tian et al. [21]. The pulmonary tissue was characterized by DAD with hyaline membrane formation and type II pneumocyte activation. They also observed fibroblastic proliferation and fibrin cluster formation. The pulmonary vessels were congested, and some alveoli lacked blood cells. The alveolar septa were thick due to fibrin and inflammatory infiltration (mononuclear cells). The liver tissue, as well as the myocardium, did not manifest pathological features traceable to COVID-19 damage.

Duarte-Neto et al. [22] performed 10 ultrasound-guided minimally invasive autopsies of patients who tested positive for SARS-CoV-2 infection. The lung parenchyma showed exudative/proliferative DAD, cytopathic respiratory epithelium damage, and an abundance of alveolar megakaryocytes. Fibrinous microthrombi were found in the alveolar arterioles, as well as in various other organ vessels (glomeruli, testis, liver, and heart). Aside from comorbidity-associated and shock-related findings, they also found perivascular mononuclear infiltration of the skin in eight cases, two cases of myositis and two cases of orchitis with diffuse small vessel endothelial changes.

An autopsy case report was published in the Revista Espanola de Patología [23] regarding a deceased 54-year-old man who was hospitalized because of dyspnea, cough, fever, and chills. The nasopharyngeal swab was positive for SARS-CoV-2. In situ sampling was performed to reduce the risk of contamination. The lungs appeared heavy, firm, and congested. Histological analysis showed both exudative and organizing DAD, platelet thrombi in small and medium vessels, slight septal thickening, and capillary congestion. There were also rare mononuclear inflammatory infiltrates and pneumocyte hyperplasia with cytopathic changes. The kidney had cortical necrosis.

In May, Magro et al. [24] reported five cases of lung and/or skin microvascular injury in patients who tested positive for SARS-CoV-2. In two cases, minimally invasive autopsies were conducted. The main histological feature was a pauci-inflammatory septal capillary injury with significant septal capillary fibrin deposition, alongside neutrophil infiltration of the septa. DAD with hyaline membranes and type II pneumocyte hyperplasia were not the main characteristics. In addition, the authors found relevant signs of systemic activation of the complement cascade, both in the lungs and skin biopsies. A notable finding was the presence of both SARS-CoV-2 spike glycoproteins and C4d and C5b-9 in the alveolar septa.

### Complete autopsies

Su et al. [25] published a case series of 26 autopsies at the beginning of April 2020, focusing mainly on renal findings. The main histological feature was acute tubule injury (ATI); in two cases, there was also acute pyelonephritis. In one of these two cases, there was evidence of inflammatory cell infiltration of an arcuate artery alongside red cell aggregation in the peritubular and glomerular capillaries. Some lymphocytic infiltrate areas were also observed. Through electron microscopy, the authors identified some viral particles in the renal epithelium and podocytes. Immunohistochemistry did not reveal specific accumulation of inflammatory cells and confirmed the presence of red cell aggregates in the microvessel lumen.

Barton and colleagues [26] reported the findings of two complete autopsies of postmortem SARS-CoV-2-positive decedents who died just before or some hours after hospital admission. While alive, both patients had fever and shortness of breath, but only one of them also had a cough. Postmortem nasopharyngeal swabs were positive for SARS-CoV-2 (rRT-PCR); in one case, the lung parenchyma swab was also positive. Postmortem radiography showed bilateral pulmonary opacities, and macroscopic examination showed heavy, red-maroon lungs. Microscopic examination revealed acute DAD with hyaline membranes and thrombi within the small pulmonary arteries in one case and foci of aspiration pneumonia in the other case. Immunohistochemistry also demonstrated the presence of CD3-, CD4-, and CD8-positive T cells, plus numerous macrophages in the patient with focal pneumonia. The decedent who showed DAD microscopically was determined to have died from COVID-19; in the other case, COVID-19 was considered an “other significant condition”.

Grimes et al. [27] described the autopsy findings of two patients who died from COVID-19 at their institution. In both cases, pulmonary thromboembolism was found, causing occlusion of the right main pulmonary artery in one case and both main pulmonary arteries in the other case. Histology confirmed this finding, and deep venous thrombosis was present in both cases. Electron microscopy showed viral inclusions within the pneumocytes.

Varga et al. [28] reported the cases of three patients with COVID-19. Autopsies were performed in two cases; the third patient was still alive, so only histology of a part of the small intestine, which was resected due to mesenteric ischemia, was performed. The aim of this study was to demonstrate endothelial damage to various organs. The lungs of the two decedents showed signs of DAD and ARDS. In all cases, lymphocytic endotheliitis was seen in various organs; in particular, in one case, the lung, heart, kidney, and liver, and the myocardium showed acute infarction, while no myocarditis was observed; in another case, there was endotheliitis of the pulmonary, heart and small bowel vessels with ischemic necrosis of the small intestine mucosa. In the patient who was still alive, the small intestine showed mucosal ischemic necrosis along with endotheliitis. One of the two decedents had undergone kidney transplantation, and the transplanted organ showed viral inclusion in the endothelial cells by electron microscopy.

The findings of twelve autopsies, including both complete and minimally invasive autopsies, of deceased COVID-19 patients were described by Bradley et al. [29]. The lungs were generally heavy and edematous; one patient also showed intraparenchymal hemorrhages, and two patients had pulmonary emboli. Histological findings were DAD at the acute and organizing stage with reactive type II pneumocytes. In two cases, acute bronchiolitis and bronchopneumonia were also observed. Viral particles within the cells of the lungs, trachea, kidney, and large intestine were observed by electron microscopy. In addition to nonspecific chronic changes of various organs, the authors reported acute tubular damage in one case and periportal lymphocytic inflammation of the liver in some cases.

Concerning central nervous system involvement, Paniz-Mondolfi et al. [30] reported the histological and electron microscopy findings of a man who was admitted to their hospital because of fever, confusion and two falls at home. No other suggestive symptoms were described, but the nasopharyngeal swab test for SARS-CoV-2 was positive. During hospitalization, the patient gradually decompensated and then died after eleven days. Viral particles were seen in the frontal lobe of his brain as well as within the brain’s endothelial cells. A real-time PCR test of the brain tissue also confirmed the presence of SARS-CoV-2.

A description of the postmortem investigations performed on the body of a 58-year-old woman who suddenly died of COVID-19 was provided by Lacy et al. [31]. Fever and dyspnea were reported in the week before death. She also had important comorbidities (type 2 diabetes mellitus, hypertension, obesity, asthma, chronic lower extremity ulcers). She was found in her apartment less than one day after her death, which occurred while she was in quarantine. A scene investigation and autopsy were performed, and COVID-19 was suspected due to her symptoms. The lungs appeared heavy, firm, and edematous, with some hemorrhage areas and thick mucus into the airways, and the mediastinal lymph nodes were dimensionally augmented. Histologically, the pulmonary parenchyma was confirmed to be edematous and presented hyaline membranes alongside mononuclear infiltrate of the alveolar septae. Desquamation of hyperplastic pneumocytes and multinucleated cells was also observed, as well as foamy macrophages. Focal alveolar hemorrhages were another microscopic finding. The other organs did not manifest peculiar alterations: the hepatic tissue was congested with central lobular paleness, and the kidneys showed global glomerular and mesangial sclerosis. Bronchi swabs, collected during autopsy, were positive, so her death was declared to be due to ARDS related to COVID-19.

Konopka et al. [32] provided a description of the postmortem lung findings of a 37-year-old man who had a history of asthma and type 2 diabetes mellitus. He was admitted to their hospital because of fever, cough, and myalgia. The swab test for SARS-CoV-2 was positive. Despite treatment, he worsened, and after nine days of hospitalization, he suddenly manifested cardiogenic collapse and died. The autopsy revealed heavy lungs with mucus within the airways, which was confirmed by histology to be paucicellular mucus. As expected, histology also showed chronic asthmatic alterations of the airways. There was also DAD, fibrinous airspace exudate, and rare fibrin thrombi within the small pulmonary vessels.

Prilutskiy et al. [33] conducted autopsies (restricted to the chest and abdomen) on four patients who died because of COVID-19. The aim of their study was to evaluate if hemophagocytosis was detectable through immunohistochemistry (staining for CD163) and so, in addition to the lung tissue, they also sampled the mediastinal and pulmonary hilar lymph nodes, liver, spleen, and bone marrow. The lungs showed acute exudative DAD. The lymph nodes were enlarged, and in three to four cases, the lymph nodes contained hemophagocytic histiocytes. Only in one case was the spleen enlarged, and red pulp hemorrhage was seen histologically alongside hemophagocytic histiocytes. In all cases, the liver did not present any peculiar findings. The bone marrow was characterized by myeloid hyperplasia, with no hemophagocytosis.

The case of a 44-year-old obese Hispanic woman who died of COVID-19 was reported by Yan et al. [34]. She was admitted with respiratory symptoms, but during hospitalization, she developed acute respiratory distress syndrome and Takotsubo cardiomyopathy, which clinicians suspected to be related to viral myocarditis. On the sixth day of hospitalization, she died from multiorgan failure. Autopsy revealed heavy lungs with signs of pleuritis and enlarged peribronchial lymph nodes. Histologically, the authors observed edema, an area of infarction, DAD with hyaline membranes, and pneumocyte cytopathic damage. Viral inclusions were observed by electron microscopy. Grossly, the right atrium appeared streaked, and the right ventricle was dilated. Histologically, the heart showed myxoid edema, myocyte hypertrophy, and focal nuclear pyknosis. Interestingly, CD45-positive lymphocytes were found in the left ventricular papillary muscle. The kidneys appeared normal on gross examination, but histologically, they had peritubular congestion and focal acute tubular damage.

A peculiar case was described by Fitzek et al. [35]: a 59-year-old German man experienced dizziness and a cough during a journey to Egypt. He was admitted to a local private clinic, but after some days of hospitalization, he died. The man’s body was then embalmed and transferred to Germany, where Fitzek and colleagues performed postmortem computed tomography (PMCT) and then an autopsy. The PMCT showed multifocal reticular consolidation alongside bilateral moderate pleural effusions. On gross examination, the lungs appeared firm and edematous. Signs of hemorrhagic tracheobronchitis were also recognized. Microscopically, there was DAD with diffuse hyaline membranes, alongside microthrombi and mild lymphocyte infiltration. The autopsy also showed congestive cardiomyopathy with cor adiposum as a pre-existing comorbidity.

Edler et al. [36] autopsied 80 deceased patients who tested positive, ante- or postmortem, for SARS-CoV-19. In four cases, COVID-19 was not correlated with death, and there were no radiological or autoptic findings indicative of active infection. For those with pneumonia, on gross examination, the lungs appeared heavy, with a mosaic-like pattern on the surface with evident capillary drawing. The pulmonary tissue appeared solidified. In 32 cases, deep vein thrombosis was present (demonstrated to be fresh by microscopy), and 17 of these patients also showed pulmonary artery embolism (nine peripheral, eight fatal fulminant). Regarding the microscopy findings, unfortunately, only twelve cases were investigated histologically at the time the results were published. In eight cases, DAD was found, characterized by the presence of active type II pneumocytes and fibroblasts in the context of exudative edema and hyaline membrane. There was also fibrosis and squamous metaplasia in advanced stages. The pulmonary vessel endothelia did not show vasculitis alterations, but the small arteries had conspicuous lymphocytes and plasma cell infiltration. In the remaining four cases, there was granulocyte focal confluent bronchopneumonia. Regarding other microscopic findings, the liver, kidneys, or intestine showed shock-related changes (four cases), as well as other chronic alterations.

The autopsy and microscopic findings of 67 cases were described in detail by Bryce et al. [37]. In summary, the lung parenchyma appeared variously altered, from patchy to diffusely consolidated, with acute DAD alongside hyaline membrane formation. Immunohistochemistry revealed thrombi within the small and medium pulmonary arteries. In 15 cases, the epicardium showed mononuclear and CD4-positive lymphocyte infiltrate, which was associated with thrombi in three cases. The kidney had acute tubular injury in six cases. Microthrombi and acute infarction of the brain were also observed.

Menter et al. [38] described the features of various organs in 21 autopsies (17 complete, and the remainder partial autopsies) of SARS-CoV-2 positive subjects. All of them had previous comorbidities (hypertension and obesity were the most common), and 14 of them took renin–angiotensin–aldosterone-system (RAAS)-modulating drugs. The lungs appeared heavy, firm, and congested; the parenchyma features were varied: some looked patchy, some had consolidations, and some others showed suppurative bronchopneumonia infiltrates. Histologically, the main feature was exudative DAD, while in eight cases, DAD was in the proliferative stage. Ten patients also had superimposed bacterial bronchopneumonia. Capillary congestion was another predominant feature, as well as edema and alveolar hemorrhage. Immunohistochemistry for fibrin was performed in eleven cases, and it showed microthrombi in the alveolar capillaries in five cases; the presence of fibrin in the capillaries was also confirmed by electron microscopy (performed in two cases). The authors also described the findings of other organs in detail: in particular, they highlighted that senile cardiac amyloidosis was four times more prevalent in these autopsies than in examinations conducted in their institute between 2018 and 2019, which was significant. Shock-related signs were common, the kidney showed diffuse acute tubular injury and, in some cases, disseminated intravascular coagulation was seen.

Remmelink and colleagues [39] described the pathological findings of 17 autopsies conducted on COVID-19 patients who died of respiratory or multiorgan failure (9 and 8, respectively). The lungs were heavy and firm with hemorrhage areas; in two cases, thrombi in the large pulmonary arteries were seen on gross examination. Histologically diffuse exudative DAD was the main feature (15/17), alongside late-stage DAD, microthrombi in small lung arteries (11/17), and lung infarcts (4/17). Regarding other organs, they found one case of ischemic enteritis and two cases of acute myocardial infarction. The kidneys were often enlarged with a pale cortex, petechiae, and the presence of hemosiderin in the lumen of the tubules. The authors also investigated the presence of SARS-CoV-2 in various tissues through immunohistochemistry (anti-SARS-nucleocapsid protein antibody stain, only lung tissue) and rRT-PCR the presence of SARS-CoV-2 in various tissues. The new coronavirus was detected in the lungs of 11/17 cases and in at least one organ of each patient. In particular, SARS-CoV-2 was also detected in the brain (9/11—not all the autopsies included brain evisceration), heart (14/17), liver (14/17), spleen (11/17), bowel (14/17), and kidney (10/17), and in 8/17 cases, the virus was in all the tested organs.

A prospective cohort study conducted by Wichmann et al. [40] collected data from twelve complete autopsies of outpatients and inpatients who tested positive for SARS-CoV-2. PMCT was performed in ten cases and showed reticular infiltrations and consolidating infiltrates; the ground-glass opacities were comparable with those on the antemortem CT images. The authors revealed that seven patients had bilateral deep vein thrombosis, four of whom also had massive pulmonary embolism, which was their cause of death. The total incidence of DVT was 58%. Grossly, the lungs appeared heavy, firm and congested, with pleurisy and patchy patterns on the surface and on the cutting surface. The gross examination of other organs did not reveal significant features, except for splenomegaly. Histological examination of the lung showed DAD (eight cases) with microvascular thrombi, capillary congestion, and edema. When bacterial superinfection was present, granulocytic infiltration was observed under the microscope. The other organs showed shock-related changes.

A research letter was published on 21 May 2020 by Schaller et al. [41] regarding the postmortem examinations of ten SARS-CoV-2-positive patients who died in a hospital setting. All of them had at least one pre-existing pathology, such as cardiovascular diseases (most frequent), chronic kidney failure, obesity, etc. The main histologic feature was different phases of DAD, involving, in particular, the middle and lower pulmonary lobes. The extrapulmonary findings were fibrosis and periportal lymphoplasmacellular infiltration of the liver, mild lymphocytic myocarditis in one case, and signs of epicarditis in another case.

Aguiar et al. [42] reported a case of a forensic autopsy of a woman found dead in her flat. She had a cough during the previous days, but the diagnosis of COVID-19 was made after death (positive tracheobronchial swab). The only known comorbidity was obesity (body mass index BMI 61.2 kg/m^2^). PMCT was performed and showed bilateral ground-glass opacities and panlobar consolidations. On gross examination, the lungs were heavy and firm and presented hemorrhagic edema. Microscopically, the lung tissue showed edema, early stage DAD with hyaline membranes, and intra-alveolar hemorrhages. Viral inclusions were not seen. In the interstitium, there were CD3-positive T cells and megakaryocytes. In addition to these pulmonary features, autopsy revealed only chronic tracheitis and hepatic microabscesses. This case is significant because the woman was young and did not receive any medical treatment. In the authors’ opinion, the cause of death was related to COVID-19 pulmonary alterations and high fever.

Fox et al. [43] collected data from ten autopsies of African American decedents. For all subjects, COVID-19 was stated as the cause of death. The lungs were heavy in all patients except for one who showed diffuse edema and firm parenchyma. Patchy hemorrhage was visible after fixation. The authors reported cardiomegaly and right ventricular dilatation as other significant macroscopic findings. Histologically, the main feature was DAD in different phases, predominantly (seven cases) between the exudative and proliferative phases. Except for one patient, all patients had evidence of pulmonary hemorrhage. Electron microscopy revealed cytomegalic type 2 pneumocytes with enlarged nuclei and eosinophilic nucleoli desquamated into the alveolar space. The decedents who did not receive ventilator support or who only received ventilator support for one day and had D-dimer elevation while alive also presented pulmonary microthrombi (two cases) with CD61 positivity. Immunohistochemistry also revealed the presence of CD4-positive and CD8-positive lymphocyte infiltration in the interstitial space and around bronchioles and blood vessels. In particular, some small vessels seemed to be surrounded by CD4-positive lymphocytes, with fibrin and platelet thrombi into the lumen. The authors highlighted the presence of CD61-positive megakaryocytes in the alveolar capillaries that seemed to produce platelets. In addition, cardiac histology showed single myocyte necrosis in the absence of proper necrosis areas or lymphocytic myocarditis.

Carsana et al. [44] described the postmortem examination findings of 38 deceased COVID-19 patients, focusing on pulmonary lesions. The lungs appeared heavy, edematous, and congested. The lung tissue histology revealed exudative and early or intermediate proliferative phases of DAD in all cases with focal features of interstitial, organizing, or acute fibrinous organizing pneumonia. In 33/38 cases, there were diffuse fibrin and platelet clots in the lumen of the peripheral small vessels of the lung. The authors stated reactive atypia and diffuse peripheral small vessel thrombosis were the characteristic histopathological findings. In addition, they also performed immunohistochemical analysis of selected cases, finding an abundance of CD45- and CD3-positive lymphocytes in the interstitial space, and CD68-positive macrophages predominantly in the alveoli. CD61-positive megakaryocytes were also abundant in the pulmonary capillaries (33/38 cases). Moreover, the lung specimens of ten cases underwent electron microscopy examination: some particles, which the authors assumed to be virions, were found within the both type 1 and type 2 pneumocytes in nine cases and were also localized in the macrophages in two cases. No viral particles were observed in the endothelial cells, despite fibrin and platelets being localized in the alveolar capillary lumen.

**Fig. 2:**
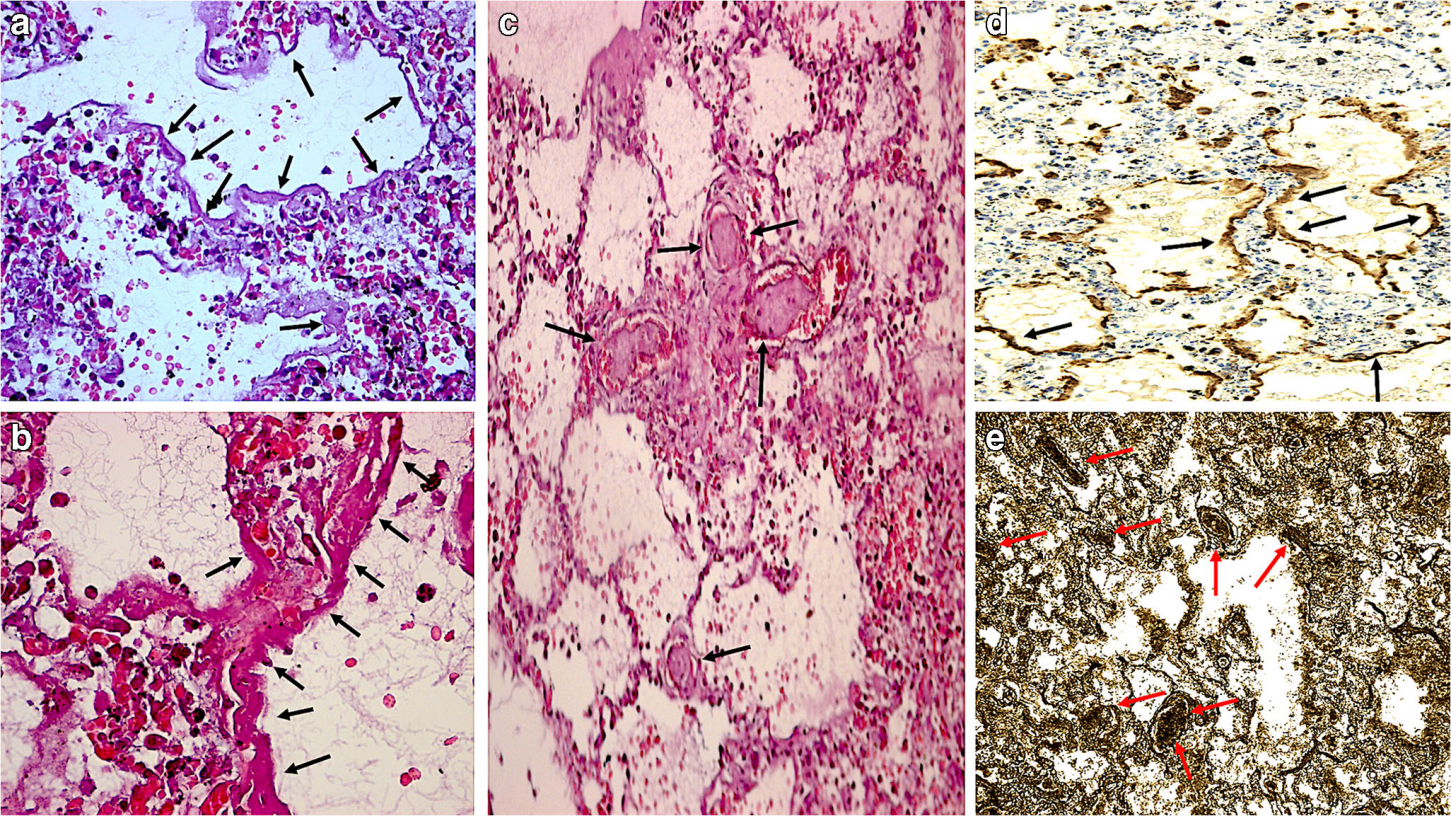
Typical histological findings described in the literature: **a**–**b** lung tissue showed edema, early stage DAD with hyaline membranes (arrows) and intraalveolar hemorrhages (H&E, × 40, × 100). **c** Lung: microvascular thrombi (arrows) (H&E, × 60), capillary congestion, and edema. DAD: **d** exudative phases (arrows) (Anti-Surfactant Protein A Antibody, × 40) with microvascular thrombi (arrows) **e** (Weigert, × 20)

Table 2 summarizes the macro- and microscopic findings as described by the cited literature. The typical histological findings described in the literature are presented in Fig. 2.

**Table 2:** Review of the literature on COVID-19 related autopsies, summary of macroscopic and microscopic findings

Minimally invasive autopsies	N° of Cases	Average age	Sex	Positive swab	Comorbidities	Cause of death	Main macroscopic pulmonary findings	Main microscopic pulmonary findings	Other findings
Xu et al	1	50 y	1 male	1	Not reported	COVID-19	-	DAD with hyaline membranes, oedema, inflammatory interstitial infiltration	Hepatic microvescicular steatosis, few cardiac inflammatory infiltrations
Zhang et al	1	72 y	1 male	1	Diabetes mellitus, hypertension	COVID-19	-	Organizing DAD, fibrinous exudate, interstitial fibrosis, chronic inflammatory infiltrates	-
Dolhnikoff et al	10	67.8 y (range 33–83)	5 male, 5 female	10 (not directly reported)	7/10 patients had comorbidities: hypertension, diabetes mellitus, ischemic heart disease, chronic obstructive pulmonary disease	COVID-19	-	Diffuse exudative and proliferative DAD, foci of alveolar haemorrhage, little lymphocytic infiltration, viral cytopathic damage of epithelium of alveoli and small airways, fibrin microthrombi in small pulmonary arterioles with a large number of megakaryocytes within pulmonary capillaries; in six cases, secondary bacterial pneumonia	Rare small fibrinous thrombi in glomerular and dermal vessels
Yao et al	3	Not reported	Not reported	3	Not reported	COVID-19	-	DAD, inflammatory interstitial infiltration (macrophages and CD4-positive T cells), hyaline thrombi in small vessels, focal haemorrhage, pulmonary interstitial fibrosis	Spleen had less lymohocytes and necrosis; features of other chronic diseases in other organs
Tian et al	4	59–81 y	3 male, 1 female	4 (not directly reported)	At least one of: CLL, cirrhosis, hypertension, diabetes, renal transplantation	COVID-19	-	DAD, hyaline membranes, type II pneumocytes activation, fibrin cluster and fibroblastic proliferation, congestion, haemorrhages	Not specific changes, sinusoidal dilatation in hepatic tissue, irregular shaped myocardium with no signs of myocarditis
Duarte-Neto et al	10	69 y (range 33–83)	5 male, 5 female	9 positive at nasopharyngeal swab and/or lung tissue	Hypertension, diabetes mellitus, chronic ischemic cardiopathy	Not reported	-	Exudative/proliferative DAD, cytopathic respiratory epithelium damage, fibrinous thrombi within alveolar arterioles (eight cases), abundance of alveolar megakaryocytes	Comorbidity-associated findings; shock signs; other findings: perivascular mononuclear infiltration of the skin in eight cases; myositis in two cases; orchitis in two cases; small vessels endothelial changes; microthrombi in various organs
Ramon y Cajal Hospital	1	54 y	1 male	1 in life nasopharyngeal swab	Hypertension, gout, migraine, obstructive sleep apnea, obesity	Not reported	Heavy, firm and congested lungs	Minimal septal thickening and capillary congestion, rare mononuclear inflammatory infiltrate, alveolar cell desquamation, pneumocyte hyperplasia with cytopathic changes; diffuse exudative DAD with hyaline membranes and organizing DAD, platelet thrombi in small and medium vessels	Kidney cortical necrosis
Magro et al	2	62 and 73 y	2 male	2 in life swab	In one case: coronary artery disease, diabetes mellitus, heart failure, prior treatment for hepatitis C virus infection, end-stage renal disease; the other case: obesity, prediabetes	Respiratory failure (not directly reported)	Congested lungs with hemorraghes	Hemorrhagic pneumonitis, septa congestion and fibrin deposition, in one case some evidence of DAD	Not reported
Complete autopsies									
Su et al	26	69 y (range 39–87)	19 male, 7 female	All	11/26 hypertension, diabetes mellitus or both, 6/26 tumor in the past, 2/26 chronic kidney disease	COVID-19	-	-	ATI, red cells aggregation into microvessels of the kidneys
Barton et al	2	59,5 y (range 42–77)	2 male	Both (post-mortem, nasopharyngeal and lung tissue swabs, + lung parenchyma for microbiologic cultures)	One of them: hypertension, cardiovascular disorder (autopsy finding), remote deep vein thrombosis and obesity (bmi 31,8); the other one: myotonic muscular dystrophy and obesity (bmi 31,3)	One case: COVID-19; coronary artery disease as “other contributing factors”; the other case: complications of hepatic cirrhosis; muscular dystrophy, aspiration pneumonia and COVID 19 as “other contributing factors”	Heavy lungs, red to maroon in color, edematous parenchyma that had diffusely firm consistency without focal lesions	In one case: DAD in the acute stage with numerous hyaline membranes and without interstitial organization; thrombi within a few small pulmonary artery branches; congestion and edema fluid focally; mucosal edema within the bronchial mucosa. In the other case: foci of acute bronchopneumonia along with rare aspirated food particles. In both immunohistochemistry showed CD3-, CD4 and CD8-positive T-lymphocytes	Not reported
Grimes et al	2	Middle-aged	2 male	Both in life nasopharyngeal swabs	Well-controlled hypertension in one case; asthma, hypertension, HIV-infection under antiretroviral therapy in the other case	COVID-19 complicated by pulmonary thromboembolism (not directly reported as the cause of death)	Pulmonary thromboembolism; bilateral pulmonary consolidation	Confirmed thromboembolism (Zhan lines); viral inclusion within the pneumocytes; fibrin inside and outside capillaries alongside platelet thrombi	In both cases deep vein thrombosis; cardiomegaly and left ventricular hypertrophy
Varga et al	3	58–71 y	2 male, 1 female	2 in life swab, for the third positive swab is not directly reported	Kidney transplantation, coronary arteries disease, hypertension in one case; hypertension, obesity, diabetes mellitus in the other case	1 mesenteric ischemia, 1 multi organ failure, 1 still alive	Not reported	DAD, vessels endothelitis	Small bowel mucosa ischemia alongside with endothelitis of various districts. Viral inclusion in the kidney transplants
Bradley et al	12	70.4 y (range 42–84)	Not reported	All in life or post-mortem	All patients had significant comorbidities: hypertension, chronic kidney diseas, obstructive sleep apnea, metabolic disease were the most common	COVID-19	Heavy, edematous lungs with intraparenchymal hemorrhages in one case. In two cases pulmonary emboli were found	DAD at acute or organizing stage with reactive type II pneumocytes; focal areas of acute bronchiolitis and bronchopneumonia in two cases. Viral particles were detected in the lungs and trachea	Non-specific chronic damage of some organs at gross examination; in one case there was also acute tubular injury; some cases showed periportal lymphocytic inflammation. Viral particles were detected in the kidney and large intestines
Paniz-Mondolfi et al	1	74 y	1 male	1 in life	Parkinson’s disease	Not reported	-	-	Viral particles in the frontal lobe and endothelial cells
Lacy et al	1	58	1 female	1 post-mortem bronchial swab	Type 2 diabetes mellitus, obesity (BMI 38), hyperlipidemia, hypertension, asthma, chronic lower extremity swelling and ulceration	COVID-19; contributory factors: type 2 diabetes mellitus, hypertension, obesity	Heavy, firm, and oedematus lungs, with some hemorrhage areas and thick mucus in the airways. Enlarged mediastinal lymph nodes	Oedema, hyaline membranes, mild mononuclear infiltrates of the septae, desquamated hyperplastic pneumocytes alongside multinucleated cells; no viral inclusion or cytopathic changes	Hepatic steatosis in a contest of congestion and central lobular pallor; mesangial sclerosis
Konopka et al	1	37 y	1 male	1 in life swab	Asthma and type 2 diabetes mellitus	COVID-19	Heavy lung with mucus within the airways	Chronic asthmatic alterations of the airways; DAD, fibrinous airspace exudate, and rare fibrin thrombi within the small pulmonary vessels	Not reported
Prilutskiy et al	4	64–91 y	3 male, 1 female	4 in life positive swabs	Not reported	COVID-19	-	Acute exudative DAD	Enlargement of mediastinal and pulmonary hilar lymph nodes, enlarged spleen only in one case
Yan et al	1	44 y	1 female	1 in life nasopharyngeal swab	Obesity, probably unrecognized systemic lupus erythematous	Multi-organ failure	Heavy lungs with signs of pleuritis and enlarged peribronchial lymph nodes	Pulmonary edema and infarction areas; acute lung injury with lymphocytic infiltrates and hyaline membranes DAD; cytopathic damage of pneumocytes alongside viral particles; perivascular lymohocytic cuffing and few lymphocytic infiltration of the vessel wall; fibrin aggregates within blood vessels	Streaking of the right atrial wall myocardial tissue and right ventricle dilatation, microscopically mild myxoid edema, myocyte hypertrophy, focal nuclear pyknosis, CD45 + lymphocytes in the left ventricular papillary muscle; focal acute tubular injury, congestion of peritubular capillaries
Fitzek et al	1	59 y	1 male	Oropharyngeal post-mortem swab	Obesity, hypertension, cardiac hypertrophy, cor adiposum (seen at external examination and/or autopsy)	Cardiorespiratory failure with other comorbidities contribution (suspicion of viral pneumonia)	Firm, edematous lungs with greyish-yellow multifocal areas, signs of hemorrhagic tracheobronchitis	DAD with diffuse hyaline membranes, vascular compression and microthrombi, edema, mild lymphocyte infiltration and inflammatory cells within the septa	Congestive cardiomyopathy with cor adiposum as pre-existing pathology
Edler et al	80	79,2 y (range 52–96)	46 male, 34 female	All (74 in life swab, 6 post-mortem nasopharyngeal or pulmonary swab)	38% overweight (13/80 cases) or obesity (17/80 cases), 85% cardiovascular disorder, 55% lung diseases, 35% CNS diseases, 34% kidney diseases, 21% diabetes mellitus, 16% carcinomas/haematological diseases In 2/80 cases no comorbidity identified	76/80 cases COVID-19 (mostly pneumonia, 8/76 of which complicated by fulminant pulmonary artery embolism, 9/76 by peripheral pulmonary artery embolism); competing causes of death were noticed in 11% of total death;in 4/80 cases virus-independent cause of death	Heavy lungs, with mosaic-like pattern on the surface and evident capillary drawing; firm and fragile tissue	In 8/12 cases DAD at different phases; lymphocytes and plasma cells infiltrate in the small arteries. In 4/12 cases, granulocyte focal confluent bronchopneumonia	deep vein thrombosis (32/80) complicated in 17/32 with vary grade of pulmonary embolism; signs of other chronic diseases; 4/12 shock changes in liver, kidneys or intestine
Bryce et al	67	69 y (range 34–94)	Not reported	All (in life nasopharyngeal swab)	Hypertension 62.7%, diabetes mellitus 40.3%, coronary artery disease 31.3%, chronic kidney disease 26.7%, asthma 17.9%, heart failure 14.9%, atrial fibrillation 13.4%, obesity 11.9%, co-infections 10.4%, cancer 7.5%, transplantation 7.5%, COPD 6%	COVID-19	Lung parenchyma appearance ranged from patchy to diffusely consolidated; in one case, multiple cavitary lesions; 4/67 cases showed pulmonary emboli in the main pulmonary arteries	Acute/exudative stage DAD (22 cases), diffuse or focal hyaline membranes, pneumocytes atypia, in two cases intranuclear inclusions. 7 cases acute pneumonia (2/7 extensive and necrotizing); 14 cases showed capillary inflammation; CD61 stains (23 cases) revealed thombi in small and medium pulmonary arteries	Viral particles and replicative structures in the lymph nodes cells; in two cases, patchy mild myocardial interstitial chronic inflammation, in 15 cases patchy epicardial mononuclear infiltrates and predominantly CD4-positive lymphocytes infiltrate associated with small vessel thrombi in three cases and with hemophagocytosisi in another case; bone marrow hemophagocytosis in four cases, spleen hemophagocytosis in nine cases; acute tubular injury in six cases; in five cases liver showed zone 3 ischemic coagulative necrosis, five cases acute outflow obstruction, portal venules thrombi in 15 cases; in six cases microthrombi and acute infarction of the brain
Menter et al	21	76 y (range 53–96)	17 male, 4 female	All (in life nasopharyngeal swab, bronchoalveolar lavage or sputum)	All cases had at least one comorbidity. Hypertension and pre-obesity/obesity were the most common; in 14/21 cases renin–angiotensin–aldosterone system-modulating drug intake; in 2 cases immunosuppressive drugs intake; 1 case presented acquired immunodeficiency	Respiratory failure	Heavy, firm and congested lungs	DAD (mainly exudative, in 8/21 cases proliferative), with superimposed bacterial bronchopneumonia in 10/21 cases; capillary congestion; oedema; alveolar haemorrhage; in 5/11 microthrombi in alveolar capillaries	senile cardiac amyloidosis (more prevalent than in past), diffuse shock signs, diffuse acute tubular injury
Remmelink et al	17	72 y (range 62–77)	12 male, 5 female	All in life swab (not directly reported)	All except two had at least one comorbidity: hypertension (10/17), diabetes mellitus (9/17), cerebrovascular disease (4/17), coronary artery disease (4/17), solid cancer (4/17)	Respiratory failure (9/17) and multi-organ failure (8/17)	Heavy, firm lungs with haemorrhage areas; thrombi in the large pulmonary arteries in 2/17 cases;	Diffuse exudative DAD (15/17), alongside late-stage DAD, microthrombi in small lung arteries (11/17), lung infarcts (4/17)	1/17 ischemic enteritis; kidneys often enlarged with pale cortex and petechiae, hemosiderin in the tubules lumen; 2/17 acute myocardial infarctions;
Wichmann et al	12	73 y (range 52–87)	9 male, 3 female	All (in life swab)	All cases had at least one comorbidity. In particular coronary heart disease (50%), COPD/asthma (25%), obesity, peripheral artery disease, diabetes mellitus 2, neurodegenerative diseases	COVID -19; in 4/12 complicated by cases massive pulmonary embolism	Heavy, firm and congested lungs, with pleurisy and patchy pattern	DAD, microvascular thromboemboli, capillary congestion, oedema; granulocytic infiltration when bacterial bronchopneumonia	7/12 cases had bilateral deep vein thrombosis, 4 of them had also massive pulmonary embolism, 3/12 fresh deep venous thrombosis and no PE; not-specific histologic findings of viral infection; shock changes
Schaller et al	10	79 y (range 64–90)	7 male, 3 female	All (at hospital admission and post-mortem)	All cases had at least one comorbidity. In particular cardiovascular disease (most frequent), chronic kidney failure, obesity	COVID -19	Not reported	Disseminated DAD at different phases, in particular in middle and lower lobes	1 case with mild lymphocytic myocarditis, 1 case with sings of epicarditis; periportal lymphoplasmacellular infiltration and fibrosis at liver histology
Aguiar et al	1	31	1 female	1 post-mortem (tracheobronchial swab)	Obesity (BMI 61,2)	COVID-19	Heavy and firm lungs, with bilateral haemorrhagic oedema	Oedema, DAD with hyaline membranes, focal intraalveolar haemorrhage, no viral inclusions or giant cells. CD3 + Tcell and megakaryocytes in the interstitium	Mild chronic tracheitis and microabscesses in the liver
Fox et al	10	Range 44–78	Not reported	All (at hospital admission)	All cases had at least one comorbidity. In particular hypertension (most common), type 2 diabetes, obesity. One patient was immunosuppressed	COVID-19	Heavy lungs (all but one) with diffuse oedema, firm tissue and patchy haemorrhage; thrombi in sections of the peripheral parenchyma	Bilateral DAD (2/10 early exudative phase, 7/10 transition to proliferative phase, 1/10 proliferative to fibrotic phase), thrombosed small vessels with associated haemorrhage	Cardiomegaly and right ventricular dilatation with single myocyte necrosis
Carsana et al	38	69 y (range 32–86)	33 male, 5 female	All (at hospital admission)	9 cases diabetes; 18 cases hypertension; 4 cases past malignancies; 11 cases cardiovascular disorders; 3 cases mild chronic obstructive pulmonary disorders (available data only for 31 subjects)	COVID-19	Pulmonary oedema and congestion with spotty involvement of the lungs	Acute or intermediate DAD in all cases, with atypical pneumocytes and diffuse peripheral small vessels thrombosis	Not reported

## Discussion

The need for postmortem information is urgent to improve patient management of mild and severe illness and therapy strategies. Nevertheless, since the initial phase of the pandemic in some countries, autopsies have been avoided [45, 46]. The first histopathologic reports of COVID-19-positive subjects were obtained from biopsies of alive patients, postmortem biopsies, surgical specimens, or transplanted organs [20, 21, 47–51]. Evidence on the safety of postmortem examinations, if properly conducted, and positive/suspected SARS-CoV-2 corpse management recommendations have appeared [52–58], and some studies about complete autopsies of deceased SARS-CoV-2-positive patients have been published.

At present, as the infection can still be considered a health care emergency in many countries, the pathophysiology of COVID-19 is only partially understood. Autopsy findings have a fundamental role, as was demonstrated in the past with other infectious diseases [14–16]. A literature review of the present evidence on the postmortem examinations of SARS-CoV-2-positive decedents highlights the main pathologic features of the disease.

The lungs generally appear heavy and edematous. Histologically, the most frequent pathological finding is both exudative and proliferative DAD in different stages, with hyaline membrane formation, inflammatory cell infiltration, and small vessel congestion. This feature bears a similarity to pulmonary damage due to SARS and MERS (Middle Eastern respiratory syndrome) [59–61]. There is also evidence that the new coronavirus SARS-CoV-2 causes endothelial dysfunction [62], which in turn could be responsible for multiorgan dysfunction [63]. Endothelial dysfunction could be explained by the expression of the receptor binding domain (RBD) on the SARS-CoV-2 surface [64], which binds ACE2 (angiotensin converting enzyme 2) receptors. ACE2 receptors are localized in many human tissues, including the endothelium [65, 66]. In addition, Magro et al. [24] found a possible correlation between the new coronavirus and complement activation.

An important feature that emerges from our data collection is the presence of thrombi in the microvessels of the lung, alongside the relatively high prevalence of deep vein thrombosis and subsequent pulmonary embolism, reported in particular by Edler et al. [36] and Wichmann et al. [40]. Some other works seem to agree with that evidence [67–69].

In our review, there is no evidence of peculiar findings in other organs correlated to COVID-19, even though Puelles et al. [70] demonstrated that the novel virus causes renal tropism, while Varga et al. [28] reported endotheliitis in various organs. In particular, although some clinical works underline possible cardiac involvement in patients with COVID-19 [3, 6, 71], from our results, myocardium damage does not seem directly attributable to SARS-CoV-2 cytopathologic effects, but further studies are needed.

There are still not enough data to draw a complete picture of the pathophysiology of SARS-CoV-2 infection. Almost all the papers considered in this review focused on pulmonary macro- and microscopic alterations, and only a few pieces of information are given about the features of other organs and systemic findings, with few exceptions. Moreover, a clear postmortem diagnostic protocol for COVID-19 has not yet been drafted. The features that allow us to count a decedent as a SARS-CoV-2 related death remain unclear. Many recommendations about the safety of autopsy procedures have been published [52–58], but to the best of our knowledge, an essential and standardized postmortem diagnostic tool for COVID-19 has not been proposed. As a consequence, the studies we reviewed show procedural differences, i.e. postmortem imaging or immunohistochemistry was conducted in all cases. The absence of uniformity among studies is a potential limitation for comparisons of data not only in our research but also in further studies. A postmortem diagnostic tool is also essential to standardizing international data concerning population studies and health care management programs.

## Conclusion

Despite attention to and investment in quantifying global burdens of disease, the diagnosis in the majority of COVID-19-related deaths currently remain unclear. This seriously limits the veracity of the disease burden estimates and, more crucially, the capacity of local health systems to respond to the disease [72]. Nonetheless, different stages of DAD and the presence of thrombi in the small arteries of the lung seem to be the main pulmonary features of the novel coronavirus disease. It is possible to identify some similarities to pulmonary damage found in other coronavirus diseases, such as SARS and MERS [60, 61]. The new coronavirus SARS-CoV-2 also seems to cause endothelial dysfunction [62], which in turn could be responsible for multiorgan dysfunction. It is important to emphasize the prevalence of deep vein thrombosis and pulmonary embolism, as well as microthrombi in the small pulmonary vessels. These features should be accurately taken into consideration by clinicians and researchers when implementing therapeutic strategies. Further autopsy studies are needed to expand this evidence and eventually highlight the pathognomonic signs of the disease. We also suggest that an international COVID-19 postmortem diagnostic tool is required not only for gathering uniform autopsy and postmortem data, but also for facilitating comparisons of population and health care studies among states.

## Key Points

1. At present, while the infection could still be considered as a health care emergency in many countries, knowing the pathophysiological mechanisms of COVID-19 is essential for correct therapy.
2. Despite attention to and investment in quantifying global burdens of disease, the diagnosis in the majority of Covid-19 related deaths remain vague.
3. A literature review of the present evidence on post-mortem examination of SARS-CoV-2 positive decedents highlights the main pathologic features of the disease.
4. A uniform COVID-19 post-mortem diagnostic protocol has not been proposed yet. In a time in which international collaboration is essential, standardized diagnostic criteria are fundamental requirements.
